# Meta-analytic studies of the glial cell marker TSPO in psychosis – a question of apples and pears? A commentary on ‘Neuroinflammation in schizophrenia: metaanalysis of in-vivo microglial imaging’ by Marques *et al*.

**DOI:** 10.1017/S003329171800421X

**Published:** 2019-02-11

**Authors:** P. Plavén-Sigray, S. Cervenka

**Affiliations:** Department of Clinical Neuroscience, Centre for Psychiatry Research, Karolinska Institutet & Stockholm Health Care Services, Stockholm County Council, Karolinska University Hospital, SE-171 76 Stockholm, Sweden

An involvement of the immune system in the pathophysiology of schizophrenia is a current topic of intensive investigation. As summarized by Marques *et al*. in this issue of *Psychological Medicine*, preliminary evidence comes from several lines of research, including genetic and epidemiological data as well as observations of increases of pro-inflammatory markers in blood and cerebrospinal fluid (Marques *et al*., 2018). However, as the authors note, in order to confirm the presence of a dysfunctional immune system in the brain, more direct methods are needed. The most established approach to examine brain immune function *in vivo* is to use positron emission tomography (PET) and radioligands that target the glial cell marker 18 kDa translocator protein (TSPO). There are at present 12 published studies that have applied this technique in psychosis patients, with seemingly inconclusive and even contradictory results (Van Berckel *et al*., 2008; Banati and Hickie, 2009; Doorduin *et al*., 2009; Bloomfield *et al*., 2015; Kenk *et al*., 2015; Coughlin *et al*., 2016; Van Der Doef *et al*., 2016; Holmes *et al*., 2016; Collste *et al*., 2017; Di Biase *et al*., 2017; Hafizi *et al*., 2017; Ottoy *et al*., 2018). However, all of these studies have employed small sample sizes (patient groups have ranged from *N* = 7 to *N* = 19): a common problem in PET neuroimaging research resulting in a low statistical power to detect patient–control differences.

One approach to overcome this limitation is to synthesize data from multiple studies using meta-analysis, which yields an estimate of an overall effect size of patient–control differences using aggregate data from published papers. In the article by Marques *et al*., the results of such analyses are reported, leading the authors to the conclusion that brain TSPO levels are elevated in patients, based mainly on studies using the first generation TSPO radioligand (R)-[^11^C]PK11195. They further conclude that there is no patient–control difference when analysing studies using the second generation TSPO radioligands [^11^C]PBR28, [^18^F]FEPPA, [^18^F]PBR111 and [^11^C]DPA713. The overall result of higher TSPO levels is in contrast to a recently published multi-center individual-participant data meta-analysis (mega-analysis) co-authored by us, partly based on the same studies (Plavén-Sigray *et al*., 2018*a*). Below we highlight some caveats that should be considered when interpreting these divergent results.

(R)-[^11^C]PK11195 was developed in the early 1990s and has been used to study glial activation in a wide range of somatic, neurological and psychiatric disorders. However, concerns regarding the low signal to noise ratio of (R)-[^11^C]PK11195 have led to the development of a series of second generation TSPO radioligands during the last decade. For (R)-[^11^C]PK11195, two main factors that contribute to the low signal-to-noise are; (1) low brain uptake (Kreisl *et al*., 2010; Kobayashi *et al*., 2017) and (2) low specific-to-background binding ratio. In PET experiments where the specific binding is blocked using a cold compound, it is possible to determine the ratio between specific and non-displaceable (background) binding, referred to as non-displaceable binding potential (BP_ND_) (Innis *et al*., 2007). For (R)-[^11^C]PK11195, BP_ND_ in healthy controls assessed in this way are in the range of 0.7–0.8, suggesting that non-displaceable binding (background signal) is proportionally larger than specific binding (target signal) (Kobayashi *et al*., 2017). This ratio is much lower than has been reported for the second-generation TSPO radioligands [^11^C]PBR28 (Owen *et al*., 2014; Plavén-Sigray *et al*., 2018c), [^11^C]DPA713 (Kobayashi *et al*., 2017) and the more recently developed [^11^C]ER176 (Ikawa *et al*., 2017). A consequence of lower biological signal is lower accuracy and reliability of the measurement (Jučaite *et al*., 2012).

In addition to the properties of the radioligand used, another factor that affects the signal-to-noise ratio of a PET outcome measure is the method of analysis. An important premise for quantification of TSPO binding is that this protein is expressed across the entire brain (Doble *et al*., 1987). This means that no region can serve as true reference for simplified quantification approaches where binding in a target region is expressed in relation to a region of non-target brain tissue. Instead, arterial blood sampling is necessary in order to model radioligand delivery to the brain (i.e. an arterial input function, AIF). Using this method, the gold standard outcome is considered to be the total distribution volume (*V*_T_), which is an estimate of radioligand binding in target tissue relative to the concentration of radioligand in plasma. In the initial two (R)-[^11^C]PK11195 studies on schizophrenia, arterial samples were collected and AIFs were established. However, instead of calculating *V*_T_, rate constants from the compartmental model was used to obtain two different types of BP: BP_P_ (denoting specific binding over plasma) (Van Berckel *et al*., 2008) and BP_ND_ (Doorduin *et al*., 2009). In the remaining (R)-[^11^C]PK11195 studies in psychosis, no AIF was collected, and BP_ND_ was calculated using the simplified reference tissue model (SRTM). Reference time-activity curves were derived either from cerebellum as a ‘pseudo-reference’ region (Holmes *et al*., 2016; Di Biase *et al*., 2017) or using the supervised cluster analysis method (Van Der Doef *et al*., 2016). Using a test–retest dataset in healthy control subjects, we recently evaluated the reliability of different measures of (R)-[^11^C]PK11195 BP_ND_, finding intraclass correlation coefficient values in the range of 0.3–0.5 (Plavén-Sigray *et al*., 2018*b*). This suggests that at least half of the variability in (R)-[^11^C]PK11195 BP_ND_ is due to measurement error. In the case of reference tissue methods, this is likely due to similar shape and magnitude of the time-activity curves in the target and reference input, yielding noisy BP_ND_ values close to zero (Plavén-Sigray *et al*., 2018*b*), an effect evident also in some of the patient studies (Holmes *et al*., 2016; Van Der Doef *et al*., 2016).

**Fig. 1.**
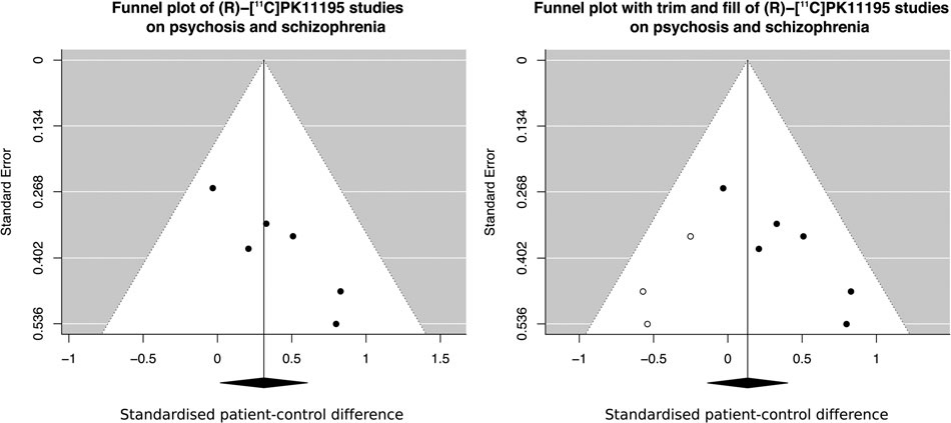
The different outcomes used by the studies included in the (R)-[11C]PK11195 meta-analysis by Marques *et al*. show little to no association with one another. This figure presents pooled data from 12 (R)-[^11^C]PK11195 examinations of healthy controls from a set of different regions (whole gray matter, thalamus, frontal cortex, hippocampus and striatum).

Low accuracy and reliability of a measurement leads to loss of sensitivity to detect true differences, as well as a higher risk for chance findings (Button *et al*., 2013; Matheson, 2018). When examining the funnel plot of the (R)-[^11^C]PK11195 meta-analysis carried out by Marques *et al*., there is a strong association between the magnitude of the patient–control difference and the measurement error of the included studies (*r* = 0.9, *p* = 0.015, Fig. 1). In other words, the larger the study, the smaller the reported effect size. This suggests that some (R)-[^11^C]PK11195 studies may have yielded inflated effect sizes, potentially due to a combination of using outcomes with low reliability, and small sample sizes. As reported by Marques *et al*., when correcting for this bias using the standard trim-and-fill method, the significant finding of elevated levels of TSPO in patients disappears. When the funnel plot displays such a shape, and the trim-and-fill correction negates an apparent effect, a general recommendation is that any non-corrected differences should be interpreted with strong caution (Duval and Tweedie, 2000; Rothstein *et al*., 2006).

A further concern with the synthesis of the (R)-[^11^C]PK11195 data by Marques *et al*. is the mixing of different outcome measures of radioligand binding. Although some between-study variation (*τ*) is allowed, a pre-condition of a random-effect meta-analysis, as performed by Marques *et al*., is that all outcomes should reflect the same underlying population effect size (Higgins *et al*., 2009):

‘The effects may be (a) assumed different and unrelated, (b) assumed different but similar […]. In the first, each study is considered in isolation from the others and meta-analysis is ruled out as an option. In the second, a random-effects model may be assumed to reflect the similarity.’

- Higgins *et al*., 2009

**Fig. 2.**
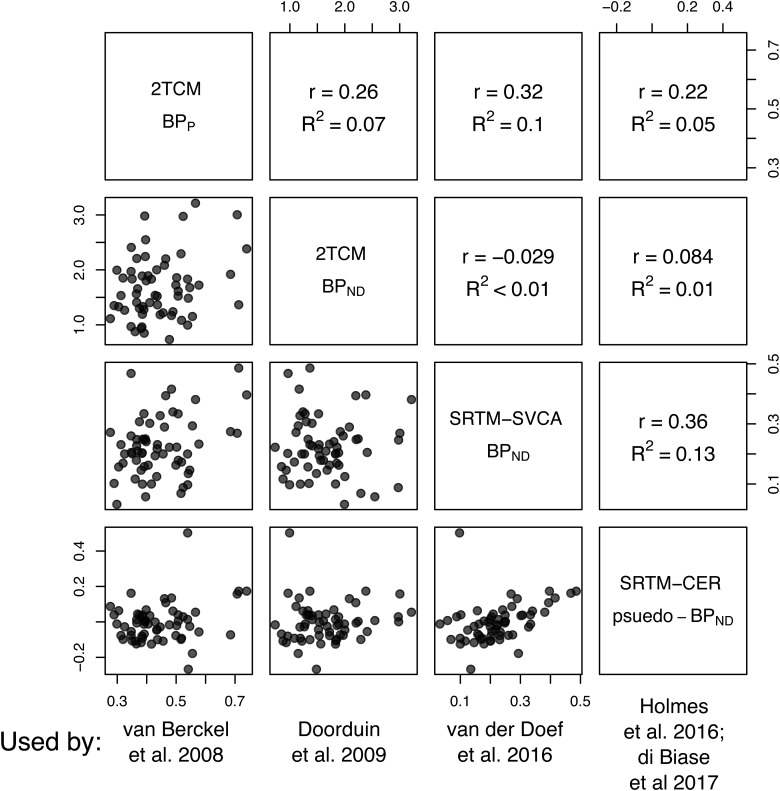
The magnitude of the effect size and the measurement error of the studies included in the (R)-[^11^C]PK11195 meta-analysis by Marques *et al*. (left figure) show a high degree of association (*r* = 0.9). Potential reasons for such a shape are publication bias or inflated effect sizes in studies with unreliable outcomes and small sample sizes, leading to an inflated overall effect size. When Marques *et al*. corrected for this bias, the difference between healthy controls and patients with psychosis or schizophrenia was no longer statistically significant (right figure).

In our test–retest paper (Plavén-Sigray *et al*., 2018*b*) we assessed whether the different (R)-[^11^C]PK11195 BP outcomes, included by Marques *et al*., are related to each other, such that criterion (b) above is fulfilled. We found low to negligible correlations between all outcomes (Fig. 2). Based on these results, it is unlikely that BP_ND_ or BP_P_ derived from the use of an AIF (Van Berckel *et al*., 2008; Doorduin *et al*., 2009), pseudo-BP_ND_ calculated from the SRTM with cerebellum (Holmes *et al*., 2016; Di Biase et al., 2017) or BP_ND_ calculated using the supervised cluster analysis method (Van Der Doef *et al*., 2016) measure the same thing. Hence, it can be argued that apples and pears and perhaps even oranges are being entered into the same meta-analytical model, calling into question the interpretability of the resulting underlying effect size.

To conclude, the low reliability and sensitivity of (R)-[^11^C]PK11195 outcomes used to examine TSPO in psychosis, caused by both radioligand characteristics and quantification methods, clearly limits the informational value of these studies. This is supported by the test–retest studies of (R)-[^11^C]PK11195 outcome measures, as well as the funnel-plot in the article by Marques *et al*. In addition, the lack of correlations between the different (R)-[^11^C]PK11195 outcome measures suggests that an important precondition of the meta-analysis model is violated. For these reasons, we do not believe that there is sufficient evidence to suggest an increase in TSPO levels in patients with psychosis or schizophrenia.

This conclusion is further supported by the second part of the meta-analysis by Marques *et al*. Here, the authors included data from studies employing second generation TSPO radioligands (Bloomfield *et al*., 2015; Kenk *et al*., 2015; Coughlin *et al*., 2016; Collste *et al*., 2017; Hafizi *et al*., 2017; Ottoy *et al*., 2018), showing no evidence in favor of higher TSPO levels in patients as compared to control subjects. We believe that this analysis has many strengths, such as (1) a higher proportion of specific signal in second-generation TSPO radioligands (Owen *et al*., 2014; Ikawa *et al*., 2017; Kobayashi *et al*., 2017; Plavén-Sigray *et al*., 2018*c*) as well as the use of a (2) homogeneous and (3) reliable outcome measure (*V*_T_) (Park *et al*., 2015; Collste *et al*., 2016; Ottoy *et al*., 2018). Moreover, we commend the authors decision not to include outcomes from these radioligands that are expressed ‘relative to tissue’ (such as distribution volume ratios), as the lack of a suitable ‘normalizing’ region makes such outcomes unreliable and prone to bias, at least in situations where there is no clear increase in the target region (Narendran and Frankle, 2016; Matheson *et al*., 2017). To summarize, we believe that the meta-analysis by Marques *et al*., including only second-generation TSPO radioligands, is both robust and of high evidential value.

The finding of no increase in *V*_T_ in psychosis or schizophrenia is in line with the mega-analysis co-authored by us, as well as by some of the authors of the Marques *et al*. study (Plavén-Sigray *et al*., 2018*a*). In fact, in this multi-center collaboration on studies using second-generation TSPO radioligands, we not only found evidence against an increase in TSPO, but also showed strong evidence in favor of *lower* TSPO in patients. Since we had access to all individual data points, it was possible to control for potential co-founders such as sex, duration of illness, symptom severity and medication effects. This is something that cannot be done in a traditional meta-analysis based on summary statistics alone, and hence allows for more robust conclusions (Tudur Smith *et al*., 2016). It should however be noted that the recently published study by Ottoy *et al*., included by Marques *et al*., was not included in our analysis. This study did not find a group difference in *V*_T_, but did find a significant age *v*. patient–control interaction. More data from clinical studies employing second generation TSPO radioligands are likely yet to come, hopefully resolving the question on whether TSPO levels are lower, or unchanged in patients with psychosis or schizophrenia.

The lack of an increase, or perhaps even the presence of a decrease in TSPO in patients, at first sight appears to contradict results from other research suggesting a pro-inflammatory state in schizophrenia. However, a closer inspection of the literature reveals that the results may be reconcilable. Importantly, there is an ongoing discussion on the lack of specificity of TSPO as a pro-inflammatory marker that deserves to be highlighted. First, we know that TSPO is not specific for microglial activation. The protein is found in astrocytes (Lavisse *et al*., 2012; Toth *et al*., 2015; Notter *et al*., 2017) as well as in vascular cells (Veronese *et al*., 2017), and even neurons (Notter *et al*., 2018). Second, animal and *in vitro* human data has challenged the widely-held view of TSPO as an exclusively pro-inflammatory marker. In a mouse model of low-grade immune activation, TSPO was found to be decreased, despite elevated levels of classical pro-inflammatory markers such as interleukin (IL)-1β and IL-6 (Notter *et al*., 2017). *In vitro* assays of human immune cells have shown that TSPO does not increase upon stimulation with the pro-inflammatory agent lipopolysaccharide (Narayan *et al*., 2017), and might even show decreased levels (Owen *et al*., 2017).

To summarize, the notion that TSPO is a microglial activation marker that represents neuroinflammation is most likely an over-simplification. Hence, evidence against increased TSPO from PET studies should not be taken as evidence against a pro-inflammatory immune state in schizophrenia, and we therefore agree with Marques *et al*. that the discussion of increased microglia activity should be kept open. However, when it comes to finding a marker that can reliably be used to detect pro-inflammatory activation in patients with psychosis as a means of patient stratification and treatment monitoring, we suggest that the search should continue elsewhere. There are a wide range of potential targets and radioligands that can be explored (Narayanaswami *et al*., 2018), and we look forward to joint efforts in translating these from validation studies in experimental settings to application in patients, thus enabling PET to realize its full potential in supporting the development of new treatment approaches for schizophrenia.
